# Interface dominated cooperative nanoprecipitation in interstitial alloys

**DOI:** 10.1038/s41467-018-06474-w

**Published:** 2018-10-01

**Authors:** Hongcai Wang, Xie Zhang, Dingshun Yan, Christoph Somsen, Gunther Eggeler

**Affiliations:** 1 Institut für Werkstoffe, Ruhr-Universität Bochum, 44801 Bochum, Germany; 2 Materials Department, University of California, Santa Barbara, CA 93106-5050 USA; 3 Institute of Mechanics, Chinese Academy of Sciences, Beijing, 100190 China

## Abstract

Steels belong to one of the best established materials, however, the mechanisms of various phase transformations down to the nano length scale are still not fully clear. In this work, high-resolution transmission electron microscopy is combined with atomistic simulations to study the nanoscale carbide precipitation in a Fe–Cr–C alloy. We identify a cooperative growth mechanism that connects host lattice reconstruction and interstitial segregation at the growing interface front, which leads to a preferential growth of cementite (Fe_3_C) nanoprecipitates along a particular direction. This insight significantly improves our understanding of the mechanisms of nanoscale precipitation in interstitial alloys, and paves the way for engineering nanostructures to enhance the mechanical performance of alloys.

## Abstract (web summary)

The specifics of nanoscale precipitation in steels remain complex. Here the authors combine high-resolution microscopy and atomistic simulations to identify a cooperative growth mechanism leading to the preferential growth of cementite nanoprecipitates along a specific crystallographic direction.

## Introduction

In recent years, there has been growing interest in understanding and tailoring the nanostructure of materials, which ultimately enables tuning material properties^1–4^. Even for extensively studied materials such as steels, there is still plenty of room for performance improvement. A number of recent publications^5–9^ have demonstrated that the key to further enhancing the mechanical performance requires engineering their nanostructures. The core concept of this new paradigm is to characterize extended defects including grain boundaries^10^, dislocations^11^, surfaces^12^, and interfaces^13^, as well as point defects such as interstitials^14^, substitutionals^15^, and vacancies^16^ etc. Even more importantly, it is of special interest to understand how these defects interact and how such interactions trigger structural evolution such as nanoscale phase transformations^17^.

A prototypical example is the precipitation of nanoscale carbides from Fe–C-based alloys^18^. The primary carbide is Fe_3_C, named cementite, which is rather stiff and brittle^19^. In contrast, the host Fe–C solid solution, called ferrite, is relatively soft and ductile. Having nanoprecipitates of cementite in a ferritic matrix allows us to overcome the tradeoff between strength and ductility in steels^20^.

However, it is by no means trivial to understand the fundamental mechanisms of this nanoscale precipitation process. First of all, it requires an accurate determination of the orientation relationships (ORs) between the precipitate and the host lattice, and the corresponding interface structures. Second, C atoms are initially homogeneously distributed in the ferrite matrix and the C concentration is only a few at.%, which is significantly lower than that in cementite (25 at.%). Hence, there should be a well-defined mechanism, which explains why and how C accumulates at the ferrite/cementite interface. Third, the ferrite matrix has a body-centered cubic (bcc) structure and C occupies octahedral interstitial sites, while the unit cell of cementite is orthorhombic and C atoms constitute a major part of the crystal structure. Thus, one needs to understand how the bcc ferrite matrix reconstructs at the interface in order to form cementite. Last but not least, the processes of C accumulation/segregation and Fe lattice reconstruction are not independent. Complex couplings between these two processes are necessary to achieve the nanoscale precipitation.

Carbide precipitation in steels has been extensively studied in the literature, correlative studies that combine atomic-scale experimental characterizations and theoretical simulations have recently been demonstrated to be an efficient approach for investigating carbide precipitation (see e.g., ref. ^21^). In this work, we combine high-resolution transmission electron microscopy (HRTEM) with atomistic simulations to unveil the nanoscale growth process of precipitated cementite from the ferrite matrix in a prototypical Fe–Cr–C alloy. We find that there is a clear driving force for C to segregate to the ferrite/cementite interface. The segregation of C atoms then cooperates with a collective reconstruction of the bcc ferrite lattice, which then naturally transforms into cementite.

## Results

### HRTEM characterization of cementite nanoprecipitates

**Fig. 1:**
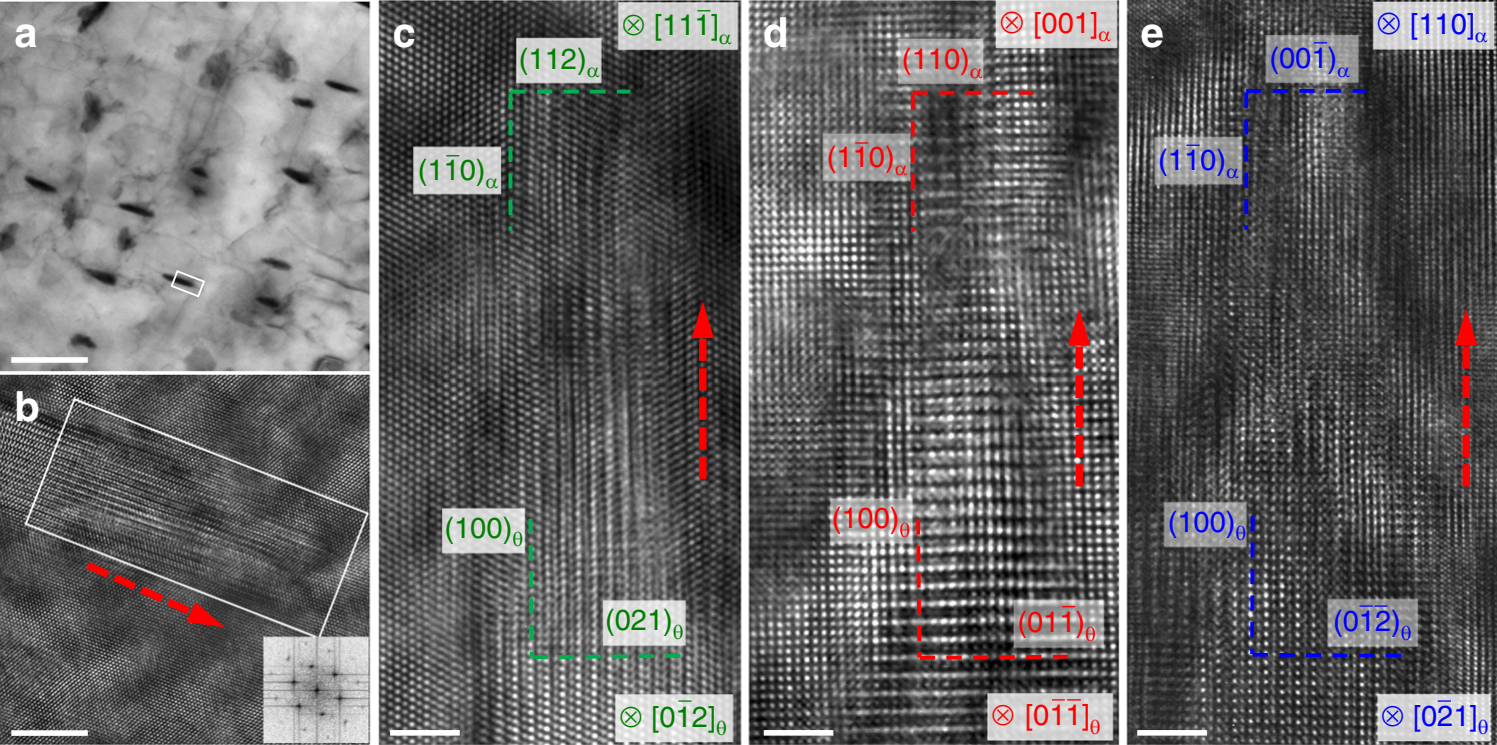
Cementite nanoprecipitates in ferrite matrix. **a** Needle-shaped nanoprecipitates of cementite (black) with preferential growth direction in a ferrite matrix (gray). **b** An HRTEM image of the cementite precipitate in **a** enclosed by a rectangle. **c**–**e** HRTEM images of the atomic structures of the advancing tip of cementite precipitates from zone directions of $[11\bar 1]_\alpha$, [001]_*α*_ and [110]_*α*_, respectively. The red dashed arrows indicate projections of the actual growth direction onto different view planes. Scale bar in **a**, 100 nm; scale bar in **b**–**e**, 2 nm

Figure 1a shows the overall microstructure of the studied Fe-10Cr-0.15C alloy (see the Methods section for details). It can be seen that needle-shaped cementite (black) precipitated from the ferrite matrix (gray). For convenience, we hereafter use *α* and *θ* to represent ferrite and cementite, respectively. An HRTEM image of the region in the rectangle in Fig. 1a is shown in Fig. 1b in the direction of $[11\bar 1]_\alpha$. It can be seen that the cementite precipitates are a few tens of nanometers long and are confined to be about 5 nanometers wide. These morphological features are indirect evidences for preferred growth directions, even though the precipitation process is not witnessed in situ. This is because the structural transformation from the parent phase to the precipitate may be accomplished by many possible mechanisms, but the driving force is the minimization of the total energy of the system. Under such circumstances, generation of interfaces that allow the best fit between the parent phase and the precipitate will be favored, since this would in turn reduce the interface energy and hence the total energy of the system. The precipitate would then grow along the easiest path to create such interfaces, giving rise to the preferred ORs. Therefore, a detailed examination of the OR between cementite and the ferrite matrix can provide vital clues to understand the precipitation mechanism in the solid state. In the past half century, a number of ORs between cementite and ferrite have been reported, most commonly the Bagaryatsky^22^, Isaichev^23^, and Pitsch-Petch^24^ ORs were observed. To determine the OR, we show the detailed atomic structure of the advancing tip of the cementite precipitate enclosed by a rectangle at a higher magnification in Fig. 1c. The cementite nanoprecipitate is found in the zone direction of $[0\bar 12]_\theta$. We perform stereographic and fast fourier transformation (FFT) analyses to determine the plane parallelism (see Supplementary Fig. 1 for details). The OR can thus be obtained as $(112)_\alpha || (021)_\theta$ and $(1\bar 10)_\alpha || (100)_\theta$ (or $[1\bar 10]_\alpha || [100]_\theta$). Figure 1d, e shows HRTEM images from two additional zone directions of [001]_α_ and [110]_α_, which are obtained by investigating another two precipitates. In these two images, the ORs are found as $[001]_\alpha || [0\bar 1\bar 1]_\theta$ ($(110)_\alpha || (01\bar 1)_\theta$ and $(1\bar 10)_\alpha || (100)_\theta$), and $[110]_\alpha || [0\bar 21]_\theta$ ($(00\bar 1)_\alpha || (0\bar 1\bar 2)_\theta$ and $(1\bar 10)_\alpha || (100)_\theta$).

**Fig. 2:**
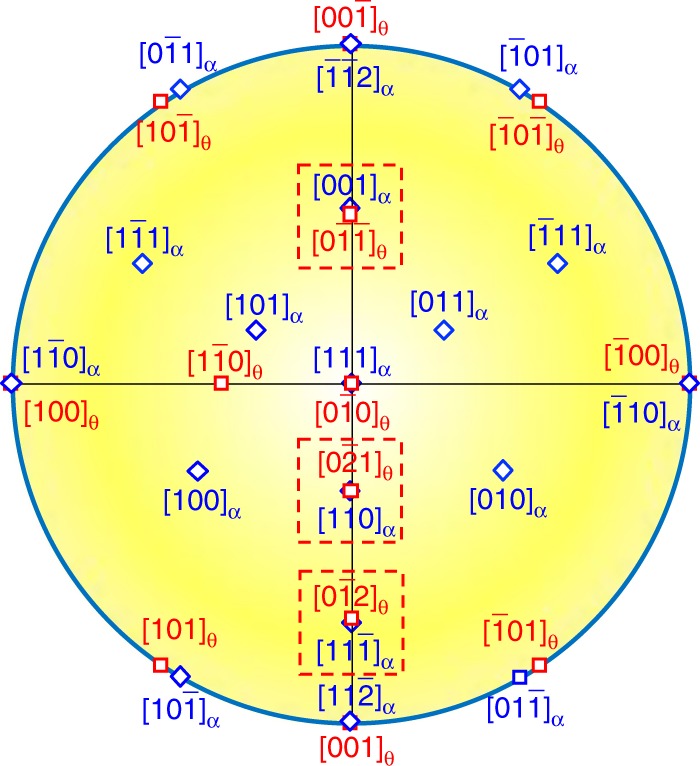
Crystallographic analysis of orientation relationship (OR) between ferrite and cementite. Stereographic projection in [111]_*α*_ orientation showing coincident or near coincident zones with cementite following the Bagaryatsky OR. The observed three variants are highlighted with square boxes. Blue diamonds are reflections from ferrite and red squares are from cementite

Figure 2 shows a stereo projection from the [111]_*α*_ direction together with cementite following the exact Bagaryatsky OR. A number of low-index coincident or near coincident directions are illustrated. It can be seen that the three images shown in Fig. 1c–e are actually three variants of the Bagaryatsky OR as highlighted by red square boxes in Fig. 1. Having established this OR, we find that the growth direction of all three precipitates is parallel to the $(1\bar 10)_\alpha$ plane. However, it is still difficult to determine the exact growth direction of the cementite nanoprecipitate, since in the HRTEM images, only two-dimensional projections from a three-dimensional object can be seen^25^. Therefore, the growth directions shown in Fig. 1 are only vector components of the real growth direction. We will clarify the growth direction in more detail later.

### Atomistic simulations of cementite nanoprecipitates

**Fig. 3:**
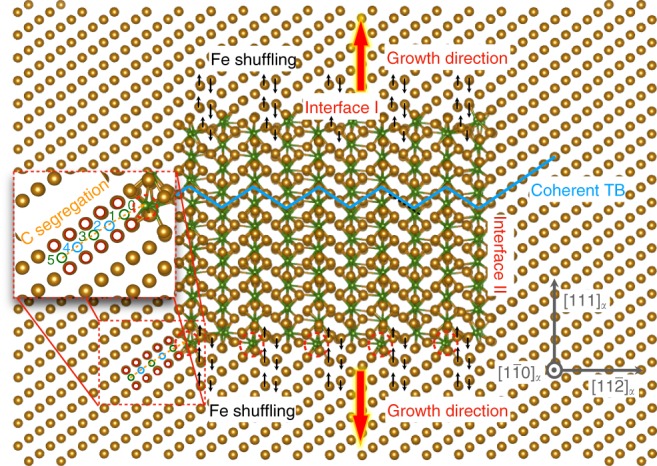
Atomic structure of a cementite nanoprecipitate in ferrite matrix. A cross-section of the atomic structure of a nanoprecipitate of cementite in a bcc ferrite matrix within the Bagaryatsky orientation relationship relaxed using the Ruda EAM potential^26^. Gold spheres represent Fe atoms and green spheres refer to C atoms. Large colored arrows show the preferential growth direction of the precipitate, which is realized by a cooperative mechanism that combines a shuffle of the Fe lattice along the ±[111]_*α*_ directions (small black arrows) and a segregation of C atoms (colored circles). The colored circles and their numbers refer to different interstitial sites for C. Light-blue lines highlight a coherent twin boundary (TB) between the bcc ferrite matrix and the cementite precipitate

In order to identify the preferred growth direction, a superlattice with a nanoprecipitate of cementite embedded in a ferrite matrix is constructed based on the exact Bagaryatsky OR. The superlattice is then relaxed using a Fe–C embedded atom method (EAM) potential^26^. Since Cr is a substitutional element and its concentration is not too high, we do not expect it to have a critical impact on the precipitation mechanism. An energy-dispersive X-ray (EDX) analysis was also conducted and shows that there is no obvious change in the Cr content across the interface between a cementite precipitate and the matrix (see Supplementary Fig. 2 for details). Moreover, introducing one more substitutional component significantly enhances the difficulty and uncertainty in EAM potential construction. We, therefore, only consider an appropriate Fe–C alloy in our atomistic simulations. A cross-section of the constructed superlattice is shown in Fig. 3. When cementite precipitates from the ferrite matrix according to the Bagaryatsky OR, three well-defined interfaces will form between ferrite and cementite. Two of them can be directly seen in Fig. 3, namely, interfaces I and II. The third one (interface III) is parallel to the visualized cross-section.

**Fig. 4:**
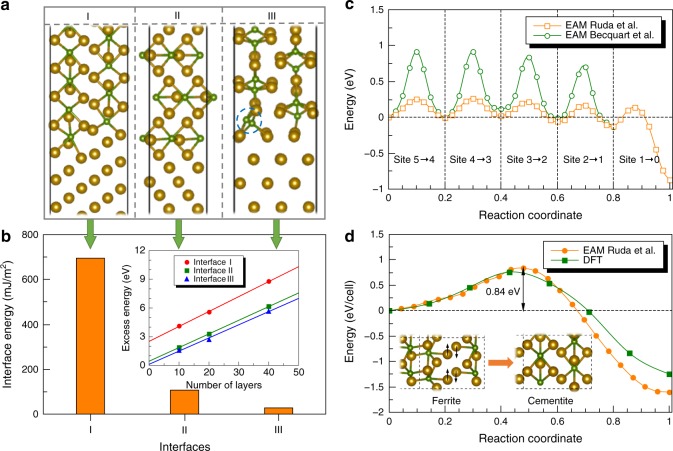
Ferrite/cementite interface structures and C segregation. **a** Atomic structures of three binary interfaces formed between cementite and ferrite during precipitating. Gold spheres represent Fe atoms and green spheres refer to C atoms. The blue dashed circle highlights the special C sites at interface III. **b** The corresponding interface energy of the three binary interfaces. The inset shows the excess energy as a function of the number of layers used for constructing the binary interfaces. **c** The energy profile of a C atom during its segregation to the interstitial site at the interface, which effectively goes through interstitial site 5 to 0 as shown in Fig. 1. **d** The minimum energy path of the bcc ferrite (with C atoms)→ cementite transition computed using both EAM potential and DFT. The inset shows the structural change from ferrite to cementite, which is realized by collective displacements of Fe (shuffling as indicated by the black arrows) and C atoms

**Fig. 5:**
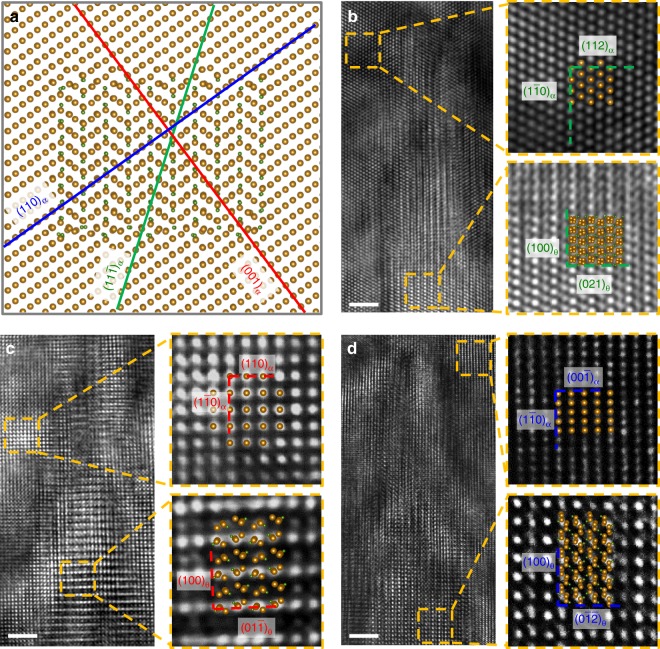
Comparison of atomic structures from theory and experiment. **a** The sectioning planes of $(11\bar 1)_\alpha$, (001)_α_ and (110)_α_ (as indicated by green, red, and blue lines, respectively) projected on $(1\bar 10)_\alpha$. **b**–**d** The atomic structures of the $(11\bar 1)_\alpha$, (001)_α_, and (110)_α_ cross-sections in the superlattice plotted on top of the experimental HRTEM images in Fig. 1(c–e). Scale bar, 2 nm

To understand the preferential growth of cementite, we first inspect the energetics of the three ferrite/cementite binary interfaces (I, II, and III). Figure 4a, b shows the structures of the three interfaces and their corresponding interface energies. It becomes immediately clear that interface I is energetically more expensive than interfaces II and III, indicating that interface I is less stable. As highlighted by the light-blue lines in Fig. 3, interface II is a coherent twin boundary (TB), which naturally explains why interface II is energetically favorable. Our close analysis reveals that interface III is favorable due to a readjustment of the positions of some C atoms (see the blue dashed circle in Fig. 4a). This indicates that at interface III there exist energetically favorable sites for C compared to bulk cementite. But since interface III is stable and these C sites at interface III are fully occupied, there would not be significant C segregation to interface III. Therefore, preferential growth will occur along the direction (the red arrow in Fig. 3) perpendicular to interface I, since the increase in the areas of interfaces II and III costs little energy. Interface I is energetically unfavorable, as a result, cementite will continuously penetrate into the ferrite matrix by moving interface I forward, which does not cost additional energy, but only needs to overcome an energy barrier. To compare with the observed growth direction components in Fig. 1, we show the simulated atomic structures from $[11\bar 1]_\alpha$, [001]_α_ and [110]_α_ in Fig. 5b–d, respectively. It can be seen that the modeled structures are in good agreement with the HRTEM observations in Fig. 1. As clarified before, the HRTEM images in Fig. 1c–e are only two-dimensional cross-sections of the three-dimensional cementite precipitates. In Fig. 5a, the projections of the sectioning planes in the experiments can be seen on the cross-section ($(1\bar 10)_\alpha$) shown in Fig. 3.

### Carbon segregation to ferrite/cementite interface

As illustrated in Fig. 3, to maintain continuous growth there must be a sufficient driving force that causes C segregation to the front of interface I. In addition, there needs to be a mechanism that reconstructs the host lattice of bcc ferrite to cooperate with the segregated C atoms in order to form cementite. As highlighted by the red dashed circles in Fig. 3, there are some C occupied sites at interface I that obviously differ from the ones inside cementite. One such site is marked site 0 in the magnified inset in Fig. 3. This type of C site periodically occurs at interface I. To understand why C prefers to segregate to interface I, we inspect the octahedral interstitial sites in bcc ferrite in the vicinity of site 0 and calculate the energy profile of C diffusion between the interstitial sites. In the inset in Fig. 3, the octahedral interstitial sites for C in bcc ferrite are marked by open circles. The three different colors (red, green, and light-blue) refer to three types of octahedral interstitial sites in a bcc host lattice, leading to tetragonal deformations along *x*, *y*, and *z* directions, respectively (see, e.g., ref. ^27^). Taking a simple diffusion path from site 5 to site 0 for example, we calculate the minimum energy path (MEP) of C diffusion using the nudged elastic band (NEB) method^28^ with the Fe–C EAM potential developed by Ruda et al.^26^. The energy profile shown in Fig. 4c provides two important insights. First, there is a driving force for C to diffuse to site 0 at the interface. As compared to C in the ferrite matrix, C at site 0 is energetically more favorable by ~0.9 eV. Second, there is a more or less constant diffusion barrier for C to diffuse from site 5 to site 0. As known from the literature, the diffusion barrier of C in bcc Fe is ~0.87 eV^27^. It means that the currently employed Fe–C EAM potential has a limitation in describing the diffusion barrier of C in bcc Fe, which is a common problem of EAM potentials. However, we use this EAM potential mainly because it gives a reliable description for the ferrite/cementite interface structure as shown in the literature^26,29^. To demonstrate this point, the energies of the structures along the MEP obtained from the Ruda EAM potential are calculated using a different EAM potential developed by Becquart et al.^30^, which is known to correctly account for the C diffusion barrier in bcc ferrite. As shown in Fig. 4c, one can see that qualitatively the diffusion energy profile is similar, but quantitatively we get now the correct diffusion barrier of C. Nevertheless, the limitation of the Becquart EAM potential is that it does not take cementite into account in the construction. We also test another commonly used Fe–C potential^31^, and observed similar interface structures and C diffusion profile to the ones from the Ruda EAM potential. Hence, we conclude that there is a clear driving force of ~0.9 eV for C to segregate to interface I, with a diffusion barrier of ~0.87 eV. In principle, the driving force has contributions from both chemical environment and local strain due to the misfit between the ferrite matrix and cementite precipitates. The existence of such misfit strain can be seen in Supplementary Fig. 3. As also recently shown by Liebscher et al.^32^, the existence of misfit strain can induce solute segregation to interfaces. However, our calculations show that the difference in the chemical potential of C between ferrite and cementite is already ~0.9 eV. Thus, in the present case the misfit strain has negligible impact on the C segregation. When cementite grows, C will continuously diffuse from the ferrite matrix to supply the transformation at the interface. Notably, the presence of strain may also lead to a deviation from stoichiometry in cementite at the interface, but it is beyond the focus of this work.

### Lattice transformation from ferrite to cementite

Now the key question is how the ferrite lattice reconstructs at the interface to accommodate the segregated C. Assuming that 25 at.% C occupying interstitial sites like site 0 has been accumulated at interface I, we identify an orthorhombic unit cell with 12 Fe atoms and 4C atoms for ferrite (dashed box) similar to the unit cell of cementite as shown by the inset in Fig. 4d. The MEP of the ferrite (with 25 at.% C) → cementite transition is then computed to understand the atomistic mechanism using again the NEB method^28^ based on both the Ruda EAM potential and density-functional theory (DFT) (see the Methods section for details). The results shown in Fig. 4d reveal that the Ruda EAM potential gives a rather similar energy profile to the one obtained by DFT, which further validates its reliability in describing the ferrite/cementite interface and the related phase transformations. An energy barrier of ~0.84 eV/cell is observed along the MEP. More importantly, our detailed analysis of the atomic displacements along the MEP reveals that the ferrite → cementite transformation mainly involves a shear-like shuffling of the Fe atoms along ±[111]_*α*_ directions as indicated by the black arrows in Fig. 2 and the inset in Fig. 4d. When Fe atoms shuffle along ±[111]_*α*_ directions, the C atoms cooperatively adjust their positions to fit into their new atomistic environment. Such cooperative displacements of the Fe and C atoms realizes the transformation from ferrite to cementite at the interface front. In fact, as we have found in our earlier studies^17^, there exists a metastable intermediate structure (MIS) during the ferrite → cementite transition. The MIS stands among bcc ferrite, fcc austenite and orthorhombic cementite, and realizes the structural transformations among the three phases by shuffling the Fe lattice along ±[111]_*α*_ directions. Therefore, combining the C segregation to interface I with the collective reconstruction of the Fe lattice, there is now a consistent picture how the nanoprecipitate of cementite grows along the direction perpendicular to interface I.

To conclude, we have identified a preferential growth direction of nanoprecipitates in a Fe–Cr–C alloy. HRTEM characterizations and atomistic simulations were used to understand the fundamental mechanisms. It was found that there exists a sophisticated cooperative mechanism that combines interstitial segregation with collective host lattice reconstruction at the interface front. This finding constitutes a critical step towards a better understanding and tailoring of nanoprecipitates in interstitial alloys, which has a direct technological impact on making ultra-high-performance alloys.

## Methods

### Sample preparation

Button-shaped with diameters of 20 mm alloy ingots were produced by arc-melting electrolytic Fe (99.99 wt.% purity), Cr (99.99 wt.% purity) and a Fe–C pre-alloy (2.15 wt.% C) under vacuum. The ingots were flipped and melted 10 times under high vacuum in the arc-melter to ensure homogeneity. The alloy was then solution treated at 1150 °C for 4 h followed by water-quenching to obtain martensitic microstructures. Sample discs of 5 mm in diameter and 0.8 mm thick were then machined from the ingot. Nanosized cementite precipitates were obtained by heating the sample discs at 10 °C/min to 270 °C in vacuum and quenched with hydrogen gas to room temperature in a TA DIL 805A/D dilatometer.

### HRTEM characterization

TEM thin foils were prepared by grinding 3 mm-diameter discs to a thickness of 50 μm, followed by twin-jet electropolishing in a TenuPol-5 (from Struers). Good thinning conditions were achieved using an electrolyte consisting of 70 vol.% methanol, 20 vol.% glycerin, and 10 vol.% perchloric acid, flow rates between 15 and 20 and voltages of 30 V at −11 °C. HRTEM imaging was performed using an FEI Tecnai Supertwin F20 equipped with a field emission gun operating at 200 kV. The zone directions of cementite nanoprecipitates were analyzed using FFT methods.

### Superlattice construction

We constructed a superlattice consisting of 5 × 5 × 5 supercell of cementite (1500 Fe atoms + 500C atoms) embedded in a bcc Fe host lattice of 186000 Fe atoms with the Bagaryatsky OR^22^ to simulate the nanoprecipitate of cementite in the ferrite matrix (see Supplementary Data 1 and 2). According to our comparison shown in Supplementary Fig. 4, the size of the cementite precipitate in our atomistic simulations is comparable with experimental one.

### Atomistic simulations

Atomistic simulations were performed using the lammps^33^ package with mainly the EAM potential developed by Ruda et al.^26^. The EAM potential by Becquart et al.^30^ is employed to inspect the diffusion barrier of C in bcc ferrite. The modified EAM (MEAM) potential by Liyanage et al.^31^ is used for cross validation. A detailed comparison of the performance of the empirical potentials is provided in Supplementary Note 1 and Supplementary Tables 1 and 2. Atomic relaxations are performed until residual forces are smaller than 10^−3^ eV/Å.

### Interface energy

As shown by Ruda et al.^26^, computing the interface energy between ferrite and cementite is non-trivial due to a linear scaling of the elastic strain energy with the number of atomic layers used in the calculations. Following the scheme proposed by Ruda et al., we first computed the excess energy of the interface as a function of the number of layers (see the inset in Fig. 4b). By extrapolating these lines to an infinitely thin interface, we could then extract the net contribution of the interface energy. Divided by the corresponding interface area, we obtained the interface energies for three binary interfaces (I, II, and III) as shown in Fig. 4b.

### NEB simulations

NEB simulations were performed using the climbing-NEB algorithm developed by Sheppard et al.^28^ as implemented in both the lammps and vasp^34^ packages. Linearly interpolated images between the initial and final configurations were used to initialize the transition path for the NEB calculations. A spring constant of −5 eV/Å^2^ was used to constrain the structural relaxation along the transition path.

### DFT calculations

DFT calculations were carried out using the vasp^34^ package. Spin-polarization was taken into account for all DFT calculations. We used the projector augmented wave (PAW)^35^ potentials within the Perdew-Burke-Ernzerhof (PBE)^36^ parametrization of the exchange-correlation functional. Based on our convergence test, we used a plane-wave energy cutoff of 400 eV and ~10,000 k-points × atoms generated from a uniform Monkhorst-Pack^37^ sampling of the Brillouin-zone in order to ensure a reliable description of the structures.

## Electronic supplementary material

Supplementary Information

Description of Additional Supplementary Files

Supplementary Data 1

Supplementary Data 2

## Data Availability

The datasets generated during the current study are available from the corresponding authors on request
