# Supplementary material for: Interface dominated cooperative nanoprecipitation in interstitial alloys
Source: Nat Commun. 2018 Oct 1;9:4017. doi: 10.1038/s41467-018-06474-w (PMC6167330; doi:10.1038/s41467-018-06474-w)
Supplement: Supplementary file 1 — Supplementary Information [file 41467_2018_6474_MOESM1_ESM.pdf]

Supplementary Information

Interface dominated cooperative nanoprecipitation in interstitial alloys

Wang *et al.*

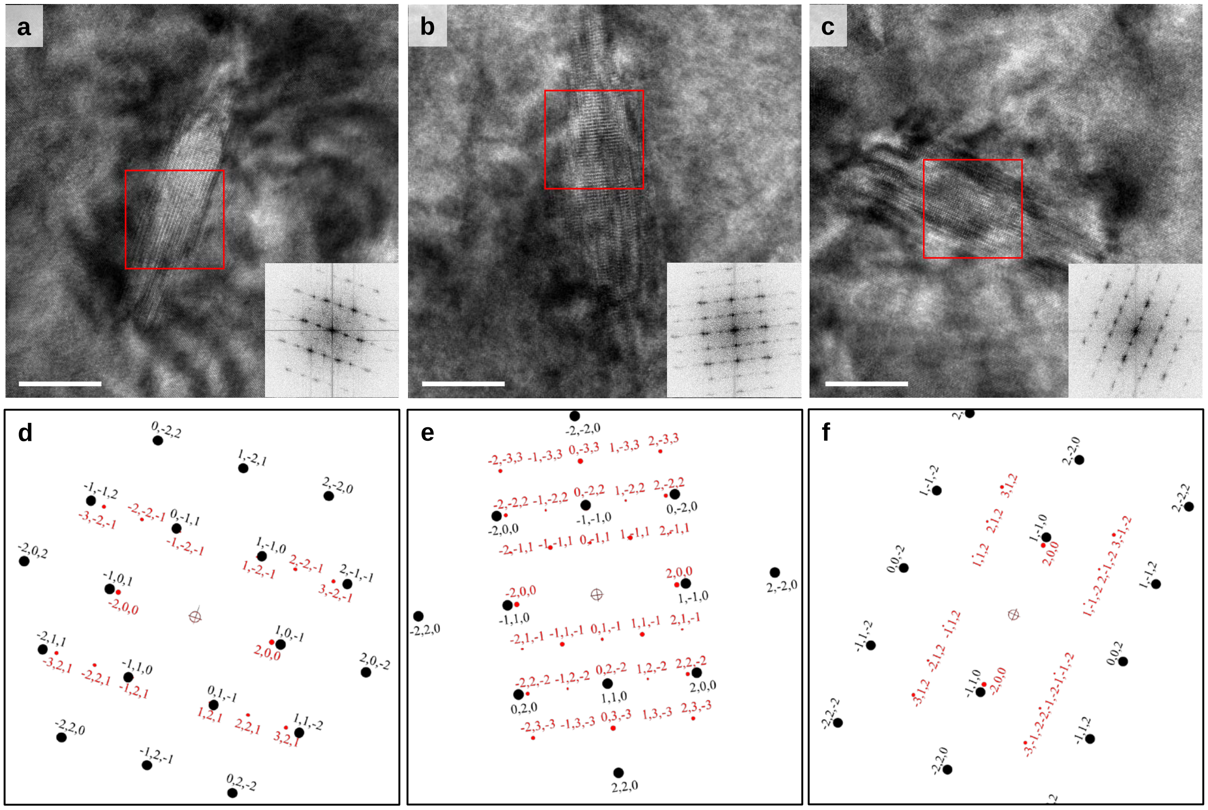

Supplementary Figure 1: **Fast Fourier Transform (FFT) analysis of the cementite orientations with respect to the ferrite matrix. a-c** Low-magnification HRTEM images show cementite nanoprecipitates in the ferrite matrix in $[11\bar{1}]_\alpha$, $[001]_\alpha$ and $[110]_\alpha$ directions. The corresponding FFT patterns of the regions highlighted by red squares in each figure are shown as insets. The cementite precipitates are in the zone direction of $[0\bar{1}2]_\theta$, $[0\bar{2}1]_\theta$ and $[0\bar{1}\bar{1}]_\theta$, respectively. **d-f** Schematic key diagrams of the three FFT patterns in (**a-c**). Black reflections are from the matrix and red from cementite. Scale bar in (**a-c**), 10 nm.

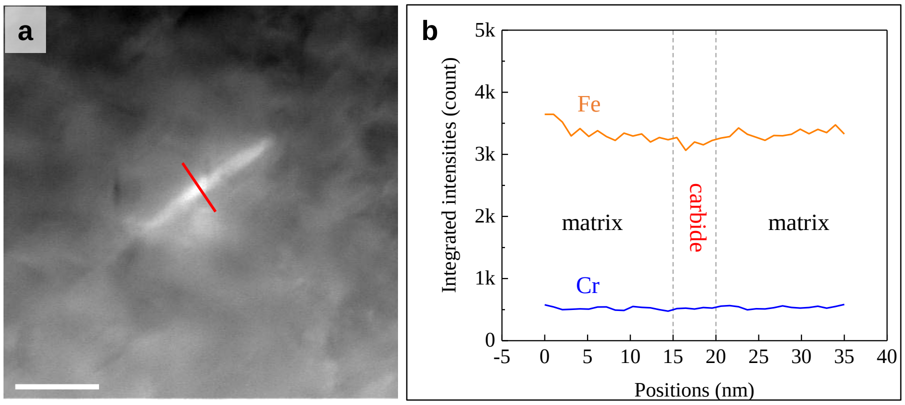

Supplementary Figure 2: **EDX analysis of the Cr distribution across interfaces between matrix and cementite.** **a** STEM image of a cementite precipitate. **b** Distribution of Fe and Cr along the red profile in (**a**). There is no significant change in the distribution of Cr content across the interface. This is because the cementite precipitation occurs under 300 °C in a time interval which is too short to allow any substantial diffusion of substitutional atoms (Cr or Fe). Scale bar in (**a**), 50 nm.

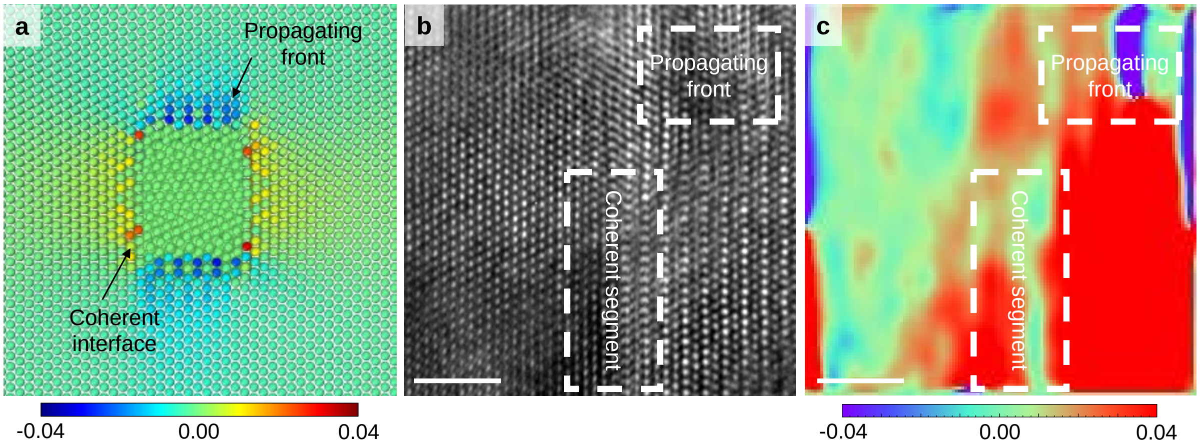

Supplementary Figure 3: Strain analysis near interfaces (marked by dashed rectangles) viewing from $[11\bar{1}]_\alpha$. **a** Strain field in the relaxed atomic structure by calculating the deviations of the Fe atoms from the ideal positions in perfect bcc Fe and show a maximum strain of about 3% at the interfaces. **b** HRTEM image of the interfaces analyzed by the GPA method. **c** Strain map generated by the GPA method using $(1\bar{1}0)$ reflection of bcc Fe. Positive strain is tensile and negative is compressive. It can be seen that the strain near the coherent interface is tensile while it is mixed with tensile and compressive at the propagating front interface. The strains are generally less than 4%. Scale bar in (**b, c**), 2 nm.

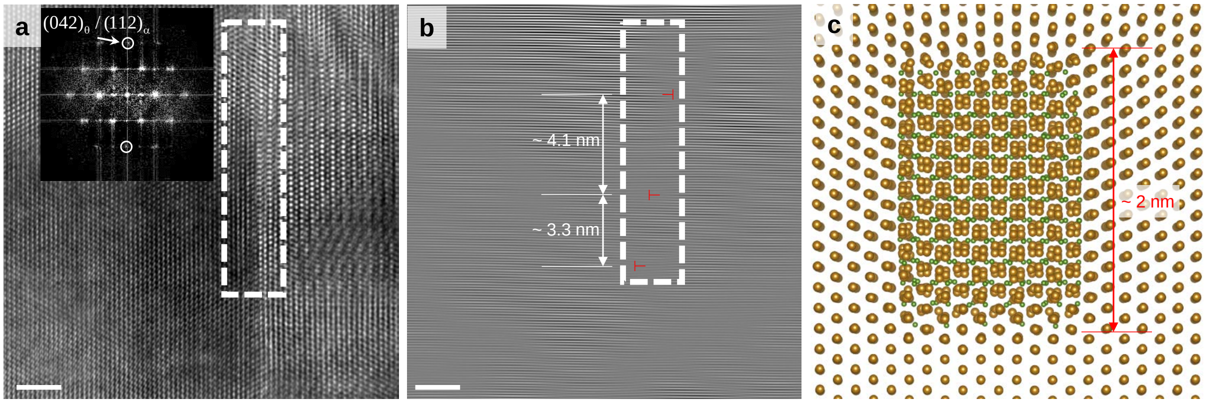

Supplementary Figure 4: **Misfit dislocation analysis of an interface viewing along** $[11\bar{1}]$ **direction.** **a** HRTEM image of the interface (marked by dashed rectangle) with the inset showing its corresponding FFT. **b** A two-dimensional lattice image from $(112)_\alpha/(042)_\theta$ reveals misfit dislocations (indicated by $\perp$) at the interface. **c** Atomic structure of the embedded cementite precipitate viewing along $[11\bar{1}]_\alpha$ direction. The cementite particle is about 2 nm long parallel to the coherent interface. Scale bar in (**a, b**), 2 nm.

Supplementary Note 1

Based on the comparisons provided in Supplementary Tables 1 and 2, the EAM potentials developed by Ruda *et al.* [1] and Becquart *et al.* [2] give similar results for the basic properties of pure bcc Fe and compares well with density-functional theory (DFT) and experiment. But the diffusion barrier of C is underestimated by the Ruda *et al.* EAM potential. The modified EAM (MEAM) potential developed by Liyanage *et al.* [3] is particularly optimized for cementite, but has a bad performance for the elastic properties of bcc Fe. The C diffusion barrier is also underestimated by the MEAM potential. The EAM potential developed by Becquart *et al.* could not stabilize the crystal structure of cementite. Overall, the EAM potential by Ruda *et al.* reasonably describe both bcc Fe and cementite. Hence, it is chosen for the major atomistic simulations in the present work.

Supplementary Table 1: Comparison of the empirical potentials with DFT and experiment for the basic properties of bcc Fe and C diffusion barrier in bcc Fe between two adjacent octahedral interstitial sites. The lattice constant a is in units of Å. The elastic constants (c_{11}, c_{12} and c_{44}) and bulk modulus (B) are in units of GPa. The diffusion barrier (ΔE_b) is in units of eV. The experimental data are collected from Refs. [4–6]

Methods	a	c_{11}	c_{12}	c_{44}	B	ΔE_b
Ruda *et al.*	2.858	284	124	115	177	0.26
Becquart *et al.*	2.855	285	124	116	178	0.90
Liyanage *et al.*	2.851	67	227	120	174	0.21
DFT	2.834	277	140	92	186	0.86
Experiment	2.867	243	138	122	173	0.87

Supplementary Table 2: Comparison of the empirical potentials with DFT and experiment for the basic properties of cementite. The lattice constants (a, b and c) are in units of Å. The elastic constants (c_{11} — c_{66}) and bulk modulus (B) are in units of GPa. The dash ”-” in the table indicates that cementite is not stable within this potential, and no meaningful elastic constants can be extracted. The DFT and experimental data are collected from Refs. [1, 3].

Methods	a	b	c	c_{11}	c_{22}	c_{33}	c_{12}	c_{13}	c_{23}	c_{44}	c_{55}	c_{66}	B
Ruda *et al.*	5.14	6.52	4.35	263	219	247	176	146	143	77	95	123	173
Becquart *et al.*	4.96	6.62	4.41	-	-	-	-	-	-	-	-	-	-
Liyanage *et al.*	5.05	6.69	4.49	322	232	326	137	170	118	17	103	64	188
DFT	5.06	6.70	4.51	385	341	316	157	162	167	13	131	131	142
Experiment	5.09	6.74	4.52	315	321	299	136	131	175	24	140	138	168

Supplementary References

[1] M. Ruda, D. Farkas, and G. Garcia, Comput. Mater. Sci. **45**, 550 (2009).
[2] C. S. Becquart, J. M. Raulot, G. Bencteux, C. Domain, M. Perez, S. Garruchet, and H. Nguyen, Comput. Mater. Sci. **40**, 119 (2007).
[3] L. S. I. Liyanage, S.-G. Kim, J. Houze, S. Kim, M. A. Tschopp, M. I. Baskes, and M. F. Horstemeyer, Phys. Rev. B **89**, 094102 (2014).
[4] C. S. Roberts, Trans. AIME **197**, 203 (1953).
[5] J. A. Rayne and B. S. Chandrasekhar, Phys. Rev. **122**, 1714 (1961).
[6] C. A. Wert, Phys. Rev. **79**, 601 (1950).
