# Supplementary material for: Interface dominated cooperative nanoprecipitation in interstitial alloys
Source: Nat Commun. 2018 Oct 1;9:4017. doi: 10.1038/s41467-018-06474-w (PMC6167330; doi:10.1038/s41467-018-06474-w)
Supplement: Supplementary file 2 — Description of Additional Supplementary Files [file 41467_2018_6474_MOESM2_ESM.pdf]

Description of Additional Supplementary Files

File Name: Supplementary Data 1
Description: Structural information of the cementite nanopreciptate in the ferrite matrix from our atomistic simulations in standard cif format.

File Name: Supplementary Data 2
Description: Structural information of the cementite nanopreciptate in the ferrite matrix from our atomistic simulations work in Excel format.
